# Book review: Advances in the understanding of gluten related pathology and the evolution of gluten-free foods; Edited by: Eduardo Arranz, Fernando Fernández Bañares, Cristina M. Rosell, Luis Rodrigo, Amado Salvador Peña

**Published:** 2015

**Authors:** Kamran Rostami, Mohammad Rostami-Nejad

**Affiliations:** 1 *Gastroenterology Unit, Milton Keynes Hospital, United Kingdom*; 2 *Gastroenterology and Liver Diseases Research Center, Research Institute for Gastroenterology and Liver Diseases, Shahid Beheshti University of Medical Sciences, Tehran, Iran*

 Some of the diagnostic, pathogenic and histological issues of gluten related disorders are still controverted. There are numerous guidelines and algorithms in the current literature, but when facing a scenario like presentation with mildly abnormal duodenal histology with positive or negative serology, most of clinician have difficulties in making a confident diagnosis. 

This book (1) is one of the few combining knowledge of the basic and clinical aspects of gluten-related disorders with the knowledge of the evolution of bread and gluten-free products. The book is written and edited by very well-known scientists who dedicated most of their life to research and supporting patients with these conditions. The knowledge will serve as a new platform in shedding more light to the dark spots in our understanding in pathogenesis of gluten related disorders (GRD) with the hope that consequently will improving the quality of life of the patients affected with these conditions. 

In this book celiac disease, dermatitis herpetiformis and a wide variety of other conditions like gluten ataxia, gluten allergy and non-celiac gluten sensitivity have been discussed. Because each chapter is written by a different specialist, there is, as in most multiauthored books, a heterogeneity in the topics that are covered. 

The first section of this book is allocated to the basic science reviewing the current knowledge on environmental, oral tolerance, treatment, genetic and immunological factors involved in celiac disease. It discusses the widely-accepted model of the pathogenesis of celiac disease which focuses on the stimulation of gluten-reactive CD4+ T cells by TG2-deamidated gluten peptides presented by HLA-DQ2/DQ8 molecules, and other non-HLA susceptibility loci that may contribute with small effects in production of inflammatory response in susceptible individuals. This section is a true enlightenment, stimulating and skilfully simplifies the matters for students, scientists in medicine, immunology and pathology.

The same section reviews the fascinating association between celiac disease and changes in the composition of intestinal microbiota that may be associated with the HLA-DQ genotype, with important implications in the pathogenesis of GRD. The imbalances in gut microbiota seem to be related to the inflammation in active phase of CD and they could also be a predisposing factor for disease development. With other word the composition of the gut microbiota could influence the release of pro-inflammatory cytokines triggered by gluten peptides. Interestingly the microbiota is not completely restored after a gluten-free diet. 

Recently, there have been some concerns raised on the gluten free products (2) and the previous studies by Sanz Y (3) argue that reductions in the intake of polysaccharides (from 117 g to 63 g on average) in healthy population after following a GFD could lead to decreased (*Bifidobacterium*, *B. longum* and *Lactobacillus*), and increased potentially unhealthy bacteria like *E. coli* and total Enterobacteriaceae. The negative effect has been attributed to the fact that polysaccharides usually reach the distal part of the colon partially undigested, and constitute one of the main energy sources for commensal components of the gut microbiota. Olivares and Sanz eloquently suggest a disruption of the delicate balance between the host and its intestinal microbiota (dysbiosis) would favour the overgrowth of opportunistic pathogens and weaken the host defences against infection and chronic inflammation via possible alterations in mucosal immunity.

The second section revises the advances in understanding the clinical spectrum of these disorders. In this sections the topic like clinical presentation, diagnostic criteria, the role of histology, extra-intestinal manifestations and the issues related to the quality of life of GRD patients have been discussed. Using γδ+ IEL are not an option available in current practice and in the same section the role of γδ+ IEL count, the detection of subepithelial tissue transglutaminase antibodies, the whole blood cytokine release assays (ELISPOT), and the tetramer test are highlighted.

The authors discuss the challenges concerned seronegative villous atrophy. Despite the current believe it might be this condition in fact doesn’t exist. What we have been calling seronegative coeliac disease might in fact be a feature of non-coeliac gluten sensitivity (NCGS). This is a new condition and despite the fact that most of patients present with minimal enteropathy (4), there is no evidence to confidently show, that enteropathy in these patients doesn’t progresses to more severe changes. The reason for this is the fact that the diagnosis of NCGS is usually prematurely excluded in patients with severe enteropathy. 

The third section explores the evolution of gluten in particular bread products that are most widely consumed in the western world. It also describes the great challenge of the elaboration of high quality gluten-free products but less expensive than the products at present available. This section on evolution of gluten-free foods will be of outmost interest to dietitians, nutritionists and patient. We would like to congratulate the authors and editors of this eminently practical book which is well suited to the needs of all scientists and clinicians who are likely to deal with patients in whom intra or extra gastrointestinal disorders develop in conjunction to gluten related disorders.
